# Prognostic significance of the albumin-to-globulin ratio for advanced urothelial carcinoma treated with pembrolizumab: a multicenter retrospective study

**DOI:** 10.1038/s41598-021-95061-z

**Published:** 2021-08-02

**Authors:** Satoru Taguchi, Taketo Kawai, Tohru Nakagawa, Yu Nakamura, Jun Kamei, Daisuke Obinata, Kenya Yamaguchi, Tomoyuki Kaneko, Shigenori Kakutani, Mayuko Tokunaga, Yukari Uemura, Yusuke Sato, Tetsuya Fujimura, Hiroshi Fukuhara, Yutaka Enomoto, Hiroaki Nishimatsu, Satoru Takahashi, Haruki Kume

**Affiliations:** 1 Department of Urology, Graduate School of Medicine, The University of Tokyo, 7-3-1 Hongo, Bunkyo-ku, Tokyo 113-8655 Japan; 2 Department of Urology, Kyorin University School of Medicine, 6-20-2 Shinkawa, Mitaka, Tokyo 181-8611 Japan; 3 Department of Urology, Teikyo University School of Medicine, 2-11-1 Kaga, Itabashi-ku, Tokyo 173-8605 Japan; 4 Department of Urology, Jichi Medical University, 3311-1 Yakushiji, Shimotsuke, Tochigi 329-0498 Japan; 5 Department of Urology, Nihon University School of Medicine, 30-1 Oyaguchi-Kamicho, Itabashi-ku, Tokyo 173-8610 Japan; 6 Division of Urology, Mitsui Memorial Hospital, 1 Kanda-izumi-cho, Chiyoda-ku, Tokyo, 101-8643 Japan; 7 Department of Urology, The Fraternity Memorial Hospital, 2-1-11 Yokozuna, Sumida-ku, Tokyo 130-8587 Japan; 8 Biostatistics Section, Department of Data Science, Center of Clinical Sciences, National Center for Global Health and Medicine, 1-21-1, Toyama, Shinjyuku-ku, Tokyo 162-8655 Japan

**Keywords:** Cancer, Immunology, Oncology, Urology

## Abstract

Although the albumin-to-globulin ratio (AGR) is a promising biomarker, no study has investigated its prognostic significance for advanced urothelial carcinoma (UC). This study conformed to the REporting recommendations for tumor MARKer prognostic studies (REMARK) criteria. We retrospectively reviewed 176 patients with advanced UC treated with pembrolizumab between 2018 and 2020. We evaluated the associations between pretreatment clinicopathological variables, including the AGR and performance status (PS), with progression-free survival, cancer-specific survival, and overall survival. The Cox proportional hazards model was used for univariate and multivariable analyses. The AGR was dichotomized as < 0.95 and ≥ 0.95 based on receiver operating characteristic curve analysis. After excluding 26 cases with missing data from the total of 176 cases, 109 (73%) patients experienced disease progression, 75 (50%) died from UC, and 6 (4%) died of other causes (median survival = 12 months). Multivariate analyses identified PS ≥ 2 and pretreatment AGR < 0.95 as independent poor prognostic factors for all endpoints. Furthermore, a prognostic risk model incorporating these two variables achieved a relatively high concordance index for all endpoints. This is the first report to evaluate the significance of AGR in advanced UC. Pretreatment AGR < 0.95 may serve as a prognostic marker for advanced UC treated with pembrolizumab.

## Introduction

Urothelial carcinoma (UC), which mainly comprises bladder cancer and upper tract UC along with urethral cancer, is intractable, particularly when advanced (locally-advanced or metastatic)^1,2^. Until recently, there was no established second-line regimen after failure of first-line platinum-based chemotherapy administered to patients with advanced UC: Patients’ median survival time following first-line chemotherapy is approximately 14 months^1–4^. However, since the mid-2010s, immune checkpoint inhibitors have been used to treat advanced UC. Notably, the KEYNOTE-045 trial found that pembrolizumab, a humanized monoclonal antibody against programmed cell death protein 1, achieves significantly better overall survival (OS) and objective response rates than cytotoxic chemotherapy (paclitaxel, docetaxel, or vinflunine) for patients with advanced UC with disease progression on or after platinum-containing chemotherapy^5^. Accordingly, pembrolizumab is currently used as an established second-line regimen^6^.

Among biomarkers of patients with UC, the neutrophil-to-lymphocyte ratio (NLR)^7^ is a well-established marker in patients with advanced UC^8–13^, including those treated with pembrolizumab^9–13^. Similarly, the albumin-to-globulin ratio (AGR) is an established marker in oncology^14–24^. Although several studies assessed AGR in patients with UC^16–24^, none evaluated its prognostic significance in the setting of “advanced UC.” Therefore, the present study assessed the prognostic significance of the AGR together with the NLR and other pretreatment clinicopathological variables of patients with advanced UC who were treated with pembrolizumab.

## Methods

### Ethical approval and informed consent

This study was approved by the Institutional Review Board (IRB) of the Graduate School of Medicine and Faculty of Medicine, The University of Tokyo (approval number: 10565), as well as that of each participating institution (IRB of Kyorin University School of Medicine; IRB of Teikyo University School of Medicine; IRB of Jichi Medical University; IRB of Nihon University School of Medicine; IRB of Mitsui Memorial Hospital; and IRB of The Fraternity Memorial Hospital). All methods were conducted in accordance with the 1964 Declaration of Helsinki and its later amendments or comparable ethical standards. Given the retrospective nature of the study, the requirement for informed consent was waived by the IRB of the Graduate School of Medicine and Faculty of Medicine, The University of Tokyo.

### Patients and study design

**Figure 1:**
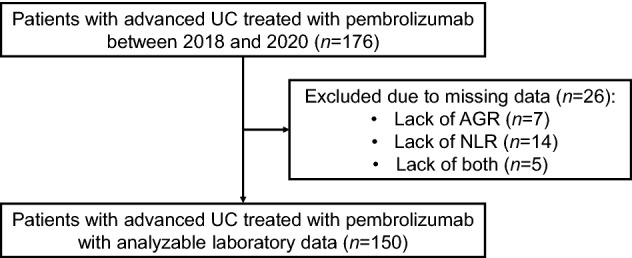
Patient selection. *AGR* albumin-to-globulin ratio, *NLR* neutrophil-to-lymphocyte ratio.

This retrospective study conformed to the REporting recommendations for tumor MARKer prognostic studies (REMARK) criteria^25^ (Supplementary Table S1 online). We reviewed the records of 176 consecutive patients with advanced (locally-advanced or metastatic) UC treated with pembrolizumab at our seven affiliate institutions (five university hospitals and two tertiary referral hospitals) between January 2018 and July 2020. Blood tests were performed within 1 month after the initiation of pembrolizumab therapy. We excluded 26 patients whose data for AGR, NLR, or both were unavailable, leaving 150 patients for the final analysis (Fig. 1). A fixed dose of pembrolizumab (200 mg per patient) was intravenously administered every 3 weeks. All patients underwent evaluations every 1–6 months that included routine blood tests, chest X-ray, and computed tomography. The patients’ charts were reviewed, and the status of each patient was assessed through office visits, telephone calls, or both.

### Pretreatment AGT, NLR, and other laboratory markers

Routine pretreatment blood tests, including serum total protein and albumin levels (g/dL), neutrophil and lymphocyte counts (cells/μL), hemoglobin (g/dL), creatinine (mg/dL), and C-reactive protein (mg/dL) were performed within 1 month before the start of pembrolizumab treatment. No patient had active infectious diseases during the blood tests. The AGR = [albumin/(total protein – albumin)]. The NLR was calculated by dividing the absolute neutrophil count by the absolute lymphocyte count. The eGFR (mL/min/1.73 m^2^) was calculated from serum creatinine levels using the formula revised for Japanese patients^26^.

### Endpoints and follow-up

We assessed the associations of pretreatment clinicopathological variables, including the AGR and NLR, with progression-free survival (PFS), cancer-specific survival (CSS), and OS. Disease progression was assessed according to the Response Evaluation Criteria in Solid Tumours (RECIST) version 1.1^27^. Follow-up started on the day of initiating pembrolizumab treatment. Follow-up information was obtained as of October 2020.

### Statistical analysis

Receiver operating characteristic (ROC) curve analysis was used to determine the optimal cutoff values of the AGR and NLR. Sensitivity, specificity, and area under the curve (AUC) were calculated using a 2 × 2 contingency table incorporating each cutoff value. The optimal cutoff value of each ratio was determined by maximization of the Youden’s index [Sensitivity − (1 − Specificity)]. The significance of the associations of the AGR with other variables were evaluated using the χ2 test or Spearman’s rank correlation coefficient. Survival curves were generated using the Kaplan–Meier method and compared using log-rank tests. The Cox proportional hazard regression model was used for univariate and multivariate analyses for PFS, CSS, and OS.

A prognostic model for predicting PFS, CSS, and OS was constructed according to independent prognostic factors detected using multivariate analysis. Harrell’s concordance index was calculated to quantify the model’s prognostic discrimination^28^. All statistical analyses, except the concordance index, were performed using JMP Pro version 14.0.0 (SAS Institute, Cary, NC, USA). Harrell’s concordance index was calculated by a biostatistician (Y.U.) using SAS version 9.4. *P* < 0.05 indicates a significant difference.

## Results

Patients’ (*n* = 150) baseline characteristics are summarized in Table 1. The median follow-up and survival times were 7.5 (interquartile range [IQR], 4–14) months and 12 (IQR, 6–29) months, respectively. Overall, 48 (32%) patients achieved an objective response, 109 (73%) experienced disease progression, 75 (50%) died of UC, and 6 (4%) died of other causes.

**Table 1:** Patients’ characteristics at the start of pembrolizumab treatment (*n* = 150).

Parameter	Value
**Age, years, median (IQR)**	71 (66–76)
**Sex, no. (%)**	
Male	111 (74)
Female	39 (26)
**ECOG PS, no. (%)**	
0	84 (56)
1	48 (32)
2	14 (9)
3	4 (3)
**Primary site, no. (%):**	
Bladder	66 (44)
Upper tract	67 (45)
Both	17 (11)
**Resection of primary site, no. (%)**	109 (73)
**Lymph node metastasis, no. (%)**	93 (62)
**Visceral metastasis, no. (%)**	106 (71)
Lung metastasis, no. (%)	59 (39)
Bone metastasis, no. (%)	30 (20)
Liver metastasis, no. (%)	27 (18)
**No. of prior regimens, no. (%)**	
1	117 (78)
2	25 (17)
3	8 (5)
**Pretreatment laboratory parameters, median (IQR)**	
AGR	1.20 (1.00–1.46)
NLR	3.34 (2.20–5.36)
Hemoglobin (g/dL)	10.9 (9.4–12.2)
eGFR (mL/min/1.73 m^2^)	48.0 (38.1–61.9)
C-reactive protein (mg/dL)	0.69 (0.15–2.50)

*AGR* albumin-to-globulin ratio, *ECOG PS* Eastern Cooperative Oncology Group Performance Status, *eGFR* estimated glomerular filtration rate, *IQR* interquartile range, *NLR* neutrophil-to-lymphocyte ratio.

ROC curve analysis identified 0.95 as the optimal discriminatory cutoff value of the AGR through maximization of Youden’s index [Sensitivity − (1 − Specificity)] for the endpoints of CSS and OS. The cutoff value (0.95) was also optimal for short-term survivals (6mo-CSS and 12mo-CSS) and suboptimal for the other endpoints (PFS, 6mo-PFS, 12mo-PFS, 6mo-OS, and 12mo-OS) (Supplementary Fig. S1 online). Similarly, the most discriminatory cutoff value of NLR = 3, given that the Youden’s index was maximized at NLR = 3.02 for CSS and OS as well as short-term survivals (6mo-PFS, 12mo-PFS, and 12mo-CSS) (Supplementary Fig. S1 online).

The χ^2^ test revealed that the pathological Eastern Cooperative Oncology Group Performance Status (ECOG PS, ≥ 2, *P* < 0.01) and bone metastasis (yes, *P* < 0.01) were significantly associated with AGR < 0.95. In contrast, the other variables (sex, primary site, resection of primary site, lymph node metastasis, lung metastasis, liver metastasis, and number of prior regimens) were not. Spearman’s rank correlation coefficient showed a strongly significant negative correlation between C-reactive protein and the AGR (ρ =  − 0.71, *P* < 0.01), as well as a weakly significant negative correlation between the NLR and the AGR (ρ =  − 0.41, *P* < 0.01) and a weakly significant positive correlation between hemoglobin and the AGR (ρ =  + 0.46, *P* < 0.01).

**Figure 2:**
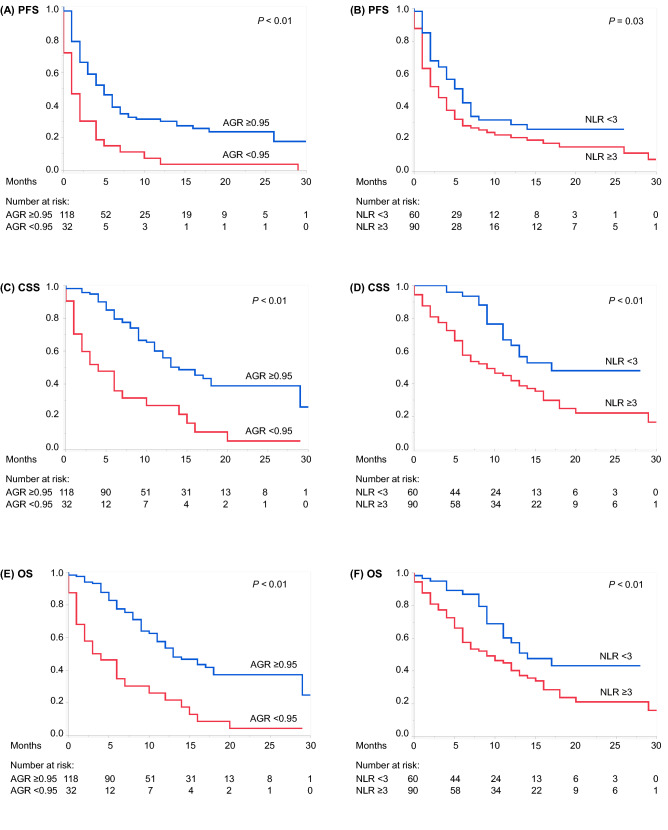
Kaplan–Meier curves depicting (**A**) PFS of patients with AGR ≥ 0.95 vs < 0.95, (**B**) PFS of patients with NLR < 3 vs NLR ≥ 3, (**C**) CSS of patients with AGR ≥ 0.95 vs < 0.95, (**D**) CSS in patients with NLR < 3 versus NLR ≥ 3, (**E**) OS of patients with AGR ≥ 0.95 vs < 0.95, and (**F**) OS of patients with NLR < 3 vs NLR ≥ 3. *AGR* albumin-to-globulin ratio, *CSS* cancer-specific survival, *NLR* neutrophil-to-lymphocyte ratio, *OS* overall survival, *PFS* progression-free survival.

Kaplan–Meier curves with log-rank tests showed significant associations of AGR < 0.95 and NLR ≥ 3 with shorter PFS, CSS, and OS (Fig. 2). Multivariate Cox proportional hazard regression analyses identified pretreatment AGR < 0.95 as an independent indicator of poor prognosis for PFS together with the following: ECOG PS ≥ 2 and liver metastasis (Table 2); CSS with ECOG PS ≥ 2 (Table 3); and OS with ECOG PS ≥ 2 (Table 4). Pretreatment NLR ≥ 3 showed a non-significant trend for shorter CSS, whereas liver metastasis did for shorter OS (both *P* = 0.08) (Tables 3 and 4).

**Table 2:** Univariate and multivariate Cox proportional hazard regression analyses of PFS.

Parameter	Cutoff	Univariate		Multivariate	
		HR (95% CI)	*P*	HR (95% CI)	*P*
Age (years)	Continuous	1.01 (0.99–1.03) per score	0.22		
Sex	Male	Reference	0.57		
	Female	0.88 (0.57–1.37)			
ECOG PS	≤ 1	Reference	< 0.01*	Reference	< 0.01*
	≥ 2	3.87 (2.27–6.62)		2.37 (1.30–4.34)	
Primary site	Bladder	Reference	0.17		
	Upper tract or both	1.30 (0.89–1.91)			
Resection of primary site	No	Reference	0.44		
	Yes	0.85 (0.56–1.28)			
Lymph node metastasis	No	Reference	0.52		
	Yes	1.14 (0.77–1.67)			
Lung metastasis	No	Reference	0.91		
	Yes	1.02 (0.70–1.50)			
Bone metastasis	No	Reference	0.06		
	Yes	1.54 (0.98–2.44)			
Liver metastasis	No	Reference	< 0.01*	Reference	< 0.05*
	Yes	2.51 (1.59–3.96)		1.83 (1.12–2.99)	
No. of prior regimens	1	Reference	0.39		
	≥ 2	1.21 (0.78–1.85)			
AGR	≥ 0.95	Reference	< 0.01*	Reference	< 0.05*
	< 0.95	2.41 (1.57–3.68)		1.82 (1.12–2.97)	
NLR	< 3	Reference	< 0.05*	Reference	0.81
	≥ 3	1.49 (1.01–2.21)		1.05 (0.68–1.63)	
Hemoglobin (g/dL)	< LLN^†^	Reference	0.42		
	≥ LLN^†^	0.81 (0.49–1.35)			
eGFR (mL/min/1.73 m^2^)	< 60	Reference	0.29		
	≥ 60	0.80 (0.53–1.21)			
C-reactive protein (mg/dL)	< 0.5	Reference	< 0.01*	Reference	0.45
	≥ 0.5	1.75 (1.19–2.59)		1.20 (0.75–1.90)	

*AGR* albumin-to-globulin ratio, *CI* confidence interval, *ECOG PS* Eastern Cooperative Oncology Group Performance Status, *eGFR* estimated glomerular filtration rate, *HR* hazard ratio, *LLN* lower limit of normal, *NLR* neutrophil-to-lymphocyte ratio, *PFS* progression-free survival.

^†^LLN of hemoglobin was set at 13.0 g/dL for men and 11.5 g/dL for women.

*Statistically significant.

**Table 3:** Univariate and multivariate Cox proportional hazard regression analyses of CSS.

Parameter	Cutoff	Univariate		Multivariate	
		HR (95% CI)	*P*	HR (95% CI)	*P*
Age (years)	Continuous	1.02 (1.00–1.05) per score	0.05		
Sex	Male	Reference	0.79		
	Female	1.07 (0.64–1.81)			
ECOG PS	≤ 1	Reference	< 0.01*	Reference	< 0.01*
	≥ 2	6.45 (3.68–11.33)		3.52 (1.76–7.05)	
Primary site	Bladder	Reference	< 0.05*	Reference	0.17
	Upper tract or both	1.62 (1.01–2.59)		1.40 (0.86–2.28)	
Resection of primary site	No	Reference	0.51		
	Yes	0.84 (0.50–1.41)			
Lymph node metastasis	No	Reference	< 0.05*	Reference	0.24
	Yes	1.75 (1.06–2.88)		1.38 (0.81–2.35)	
Lung metastasis	No	Reference	0.70		
	Yes	1.10 (0.69–1.75)			
Bone metastasis	No	Reference	< 0.05*	Reference	0.32
	Yes	1.92 (1.11–3.34)		1.35 (0.74–2.46)	
Liver metastasis	No	Reference	< 0.01*	Reference	0.18
	Yes	2.52 (1.49–4.26)		1.51 (0.82–2.78)	
No. of prior regimens	1	Reference	0.30		
	≥ 2	1.31 (0.79–2.16)			
AGR	≥ 0.95	Reference	< 0.01*	Reference	< 0.05*
	< 0.95	3.19 (1.96–5.21)		2.19 (1.19–4.04)	
NLR	< 3	Reference	< 0.01*	Reference	0.08
	≥ 3	2.48 (1.44–4.26)		1.70 (0.94–3.07)	
Hemoglobin (g/dL)	< LLN^†^	Reference			
	≥ LLN^†^	0.58 (0.30–1.13)	0.11		
eGFR (mL/min/1.73 m^2^)	< 60	Reference	0.35		
	≥ 60	0.78 (0.46–1.32)			
C-reactive protein (mg/dL)	< 0.5	Reference	< 0.01*	Reference	0.48
	≥ 0.5	2.31 (1.41–3.76)		1.24 (0.69–2.23)	

*AGR* albumin-to-globulin ratio, *CI* confidence interval, *CSS* cancer-specific survival, *ECOG PS* Eastern Cooperative Oncology Group Performance Status, *eGFR* estimated glomerular filtration rate, *HR* hazard ratio, *LLN* lower limit of normal, *NLR* neutrophil-to-lymphocyte ratio.

^†^LLN of hemoglobin was set at 13.0 g/dL for men and 11.5 g/dL for women.

*Statistically significant.

**Table 4:** Univariate and multivariate Cox proportional hazard regression analyses of OS.

Parameter	Cutoff	Univariate		Multivariate	
		HR (95% CI)	*P*	HR (95% CI)	*P*
Age (years)	Continuous	1.02 (1.00–1.05) per score	0.05		
Sex	Male	Reference	0.90		
	Female	0.97 (0.58–1.62)			
ECOG PS	≤ 1	Reference	< 0.01*	Reference	< 0.01*
	≥ 2	5.64 (3.23–9.83)		3.53 (1.85–6.74)	
Primary site	Bladder	Reference	< 0.05*	Reference	0.12
	Upper tract or both	1.63 (1.04–2.57)		1.44 (0.90–2.28)	
Resection of primary site	No	Reference	0.60		
	Yes	0.87 (0.53–1.44)			
Lymph node metastasis	No	Reference	0.12		
	Yes	1.44 (0.91–2.29)			
Lung metastasis	No	Reference	0.58		
	Yes	1.14 (0.73–1.77)			
Bone metastasis	No	Reference	0.05		
	Yes	1.71 (0.99–2.95)			
Liver metastasis	No	Reference	< 0.01*	Reference	0.08
	Yes	2.59 (1.57–4.27)		1.65 (0.94–2.90)	
No. of prior regimens	1	Reference	0.52		
	≥ 2	1.18 (0.72–1.94)			
AGR	≥ 0.95	Reference	< 0.01*	Reference	< 0.01*
	< 0.95	3.18 (1.98–5.09)		2.60 (1.48–4.59)	
NLR	< 3	Reference	< 0.01*	Reference	0.37
	≥ 3	1.95 (1.19–3.18)		1.28 (0.75–2.19)	
Hemoglobin (g/dL)	< LLN^†^	Reference	0.07		
	≥ LLN^†^	0.54 (0.28–1.04)			
eGFR (mL/min/1.73 m^2^)	< 60	Reference	0.72		
	≥ 60	0.92 (0.56–1.49)			
C-reactive protein (mg/dL)	< 0.5	Reference	< 0.01*	Reference	0.57
	≥ 0.5	2.04 (1.28–3.23)		1.17 (0.67–2.05)	

*AGR* albumin-to-globulin ratio, *CI* confidence interval, *ECOG PS* Eastern Cooperative Oncology Group Performance Status, *eGFR* estimated glomerular filtration rate, *HR* hazard ratio, *LLN* lower limit of normal, *NLR* neutrophil-to-lymphocyte ratio, *OS* overall survival.

^†^LLN of hemoglobin was set at 13.0 g/dL for men and 11.5 g/dL for women.

*Statistically significant.

**Figure 3:**
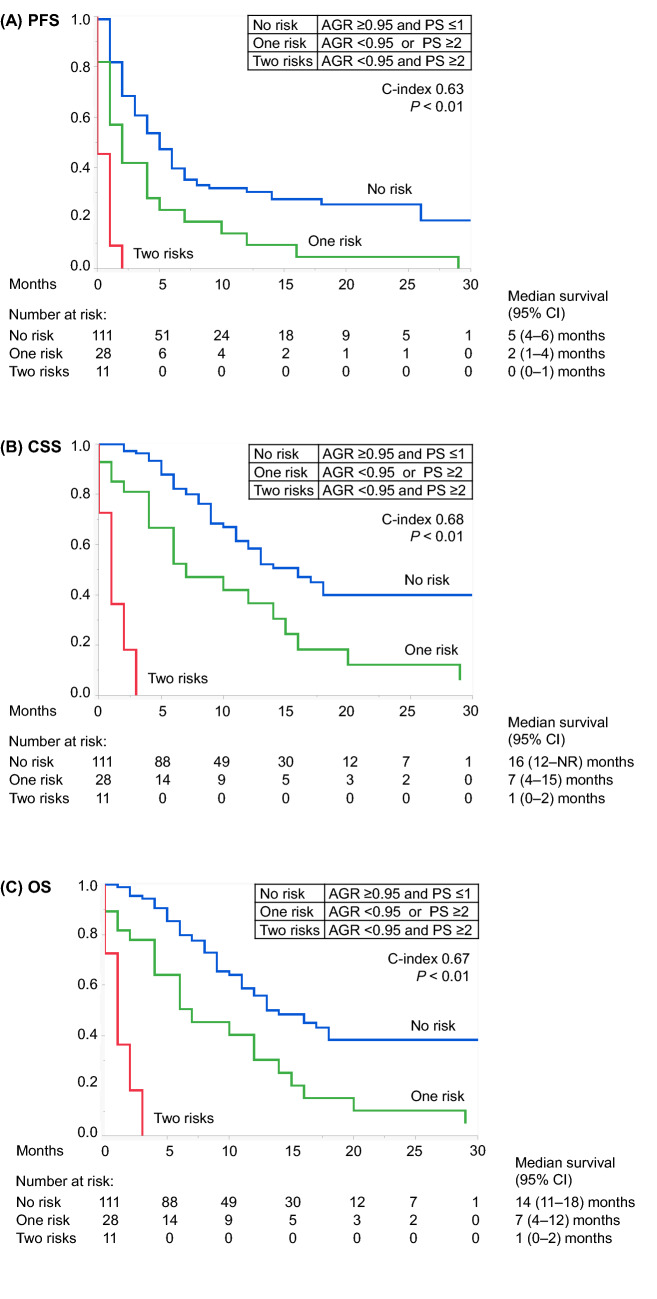
Kaplan–Meier curves depicting (**A**) PFS, (**B**) CSS, and (**C**) OS according to the prognostic risk model (no risk, AGR ≥ 0.95 and PS ≤ 1; one risk, AGR < 0.95 or PS ≥ 2; and two risks, AGR < 0.95 and PS ≥ 2). *AGR* albumin-to-globulin ratio, *CI* confidence interval, *CSS* cancer-specific survival, *NR* not reached, *OS* overall survival, *PFS* progression-free survival, *PS* performance status.

A prognostic risk model designed to predict PFS, CSS, and OS was developed according to the two shared risk factors in the multivariate analyses for all endpoints as follows: no risk, AGR ≥ 0.95 and PS ≤ 1; one risk, AGR < 0.95 or PS ≥ 2; and two risks, AGR < 0.95 and PS ≥ 2. A significant difference was found among the survival profiles of the three risk groups (Fig. 3). The Harrell's concordance indices of this model were PFS, 0.63; CSS, 0.68; and OS, 0.67.

## Discussion

To our knowledge, this study is the first study to assess the prognostic significance of the AGR in patients with advanced UC. Here we analyzed a multi-institutional cohort of 150 patients with advanced UC patients treated with pembrolizumab. We found that pretreatment AGR < 0.95 and ECOG PS ≥ 2 were independent predictors of PFS, CSS, and OS. Furthermore, we developed a prognostic risk model incorporating these two variables, which classified patients into three risk groups with significantly different PFS, CSS, and OS values. The model achieved a relatively high Harrell’s concordance index for all study endpoints.

Although the concept of AGR was introduced long ago, it was not applied to oncology until the 2010s^14,15^. Several studies assessed the prognostic significance of the AGR of patients with UC^16–24^ in settings of bladder cancer treated with radical cystectomy^16,17^, non-muscle-invasive bladder cancer^18^, and upper tract UC treated with radical nephroureterectomy^19–24^. Most of these studies^16–23^ demonstrate the utility of the AGR as a readily available predictive biomarker for patients with UC. To our knowledge, the present study is the first to demonstrate the potential utility of the AGR in the setting of advanced UC, and thus adds further evidence in this field.

The link between a low AGR and poor outcomes of patients with cancer is not fully established but may be explained in general terms as follows^23,29^: First, previous studies show that poor nutritional status or hypoalbuminemia is a predictor of poor prognosis for patients with cancer^29^. Second, chronic inflammation involving serum globulins is required for tumor proliferation, immune evasion, and metastasis. Evidence indicates that serum globulins secreted by tumor-related cells promote tumor development, immunosuppression, and metastasis^29^. Third, a low AGR may thus more sensitively reflect the degree of poor nutritional status (hypoalbuminemia) and tumor progression (hyperglobulinemia) than either measure alone, and may therefore serve as a highly significant prognostic biomarker^23^.

Similar to the concept of the AGR (i.e. use of a ratio), the NLR was developed in 2001 by Roman Zahorec^7^ and has subsequently been investigated in oncology. Numerous studies show an association with an increased NLR with worse outcomes of certain malignancies including UC^8–13^. The NLR sensitively reflects the degree of tumor progression; this is because both an increased neutrophil-dependent inflammatory reaction and a decreased lymphocyte-mediated anti-tumor immune response contribute to the elevation of the NLR^8,30^.

We previously reported the prognostic significance of the NLR using a multi-institutional cohort of 185 patients with advanced UC undergoing first-line chemotherapy^8^. Pretreatment NLR ≥ 3 was identified as an independent predictor of CSS and OS together with ECOG PS ≥ 2 and liver metastasis, whereas the AGR was not evaluated^8^. In the present study, pretreatment NLR ≥ 3 was significantly associated with all endpoints of PFS, CSS, and OS on univariate analyses (Tables 2, 3, 4), and showed a non-significant trend for shorter CSS on a multivariate analysis (*P* = 0.08) (Table 3). These data indicate that the NLR may still serve as a valid biomarker in the setting of later-line pembrolizumab.

We further reported the critical impact of liver metastasis on worse outcomes in the said study^8^. In the present study, liver metastasis was significantly associated with all endpoints of PFS, CSS, and OS on univariate analyses (Tables 2, 3, 4), and was identified as an independent predictor of shorter PFS (Table 2) with showing a non-significant trend for shorter OS (*P* = 0.08) (Table 4) on multivariate analyses. Although liver metastasis was not incorporated into the final risk model applied here, it undoubtedly serves as an essential prognostic marker for advanced UC, even in the era of immune checkpoint inhibitors.

The limitations of this study include its retrospective design and the limited number of patients. Further studies with larger populations are required to validate our results.

In conclusion, pretreatment AGR < 0.95 may serve as a prognostic marker for patients with advanced UC treated with pembrolizumab. Our newly developed prognostic risk model, including pretreatment AGR and ECOG PS, may serve as an excellent discriminator of survival.

## Supplementary Information

Supplementary Figure S1.

Supplementary Table S1.

## Data Availability

The datasets generated during and/or analyzed during the current study are available from the corresponding author upon reasonable request.

## Abbreviations

AGR: Albumin-to-globulin ratio
AUC: Area under the curve
CI: Confidence interval
CSS: Cancer-specific survival
ECOG PS: Eastern Cooperative Oncology Group Performance Status
eGFR: Estimated glomerular filtration rate
HR: Hazard ratio
IQR: Interquartile range
IRB: Institutional Review Board
LLN: Lower limit of normal
NLR: Neutrophil-to-lymphocyte ratio
OS: Overall survival
PFS: Progression-free survival
RECIST: Response Evaluation Criteria in Solid Tumours
REMARK: REporting recommendations for tumor MARKer prognostic studies
ROC: Receiver operating characteristic

## References

[1] Taguchi S, Nakagawa T, Hattori M (2013). Prognostic factors for metastatic urothelial carcinoma undergoing cisplatin-based salvage chemotherapy. Jpn. J. Clin. Oncol..

[2] Taguchi S, Nakagawa T, Uemura Y (2016). Validation of major prognostic models for metastatic urothelial carcinoma using a multi-institutional cohort of the real world. World J. Urol..

[3] von der Maase H, Hansen SW, Roberts JT (2000). Gemcitabine and cisplatin versus methotrexate, vinblastine, doxorubicin, and cisplatin in advanced or metastatic bladder cancer: Results of a large, randomized, multinational, multicenter, phase III study. J. Clin. Oncol..

[4] von der Maase H, Sengelov L, Roberts JT (2005). Long-term survival results of a randomized trial comparing gemcitabine plus cisplatin, with methotrexate, vinblastine, doxorubicin, plus cisplatin in patients with bladder cancer. J. Clin. Oncol..

[5] Bellmunt J, de Wit R, Vaughn DJ (2017). Pembrolizumab as second-line therapy for advanced urothelial carcinoma. N. Engl. J. Med..

[6] National Comprehensive Cancer Network. *NCCN Clinical Practice Guidelines in Oncology: Bladder Cancer (Version 2.2020)*. https://www.nccn.org/professionals/physician_gls/pdf/bladder.pdf. Accessed 8 Nov 2020.

[7] Zahorec R (2001). Ratio of neutrophil to lymphocyte counts–rapid and simple parameter of systemic inflammation and stress in critically ill. Bratisl. Lek. Listy..

[8] Taguchi S, Nakagawa T, Matsumoto A (2015). Pretreatment neutrophil-to-lymphocyte ratio as an independent predictor of survival in patients with metastatic urothelial carcinoma: A multi-institutional study. Int. J. Urol..

[9] Ogihara K, Kikuchi E, Shigeta K (2020). The pretreatment neutrophil-to-lymphocyte ratio is a novel biomarker for predicting clinical responses to pembrolizumab in platinum-resistant metastatic urothelial carcinoma patients. Urol. Oncol..

[10] Shimizu T, Miyake M, Hori S (2020). Clinical impact of sarcopenia and inflammatory/nutritional markers in patients with unresectable metastatic urothelial carcinoma treated with pembrolizumab. Diagnostics.

[11] Etani T, Naiki T, Sugiyama Y (2020). Low geriatric nutritional risk index as a poor prognostic marker for second-line pembrolizumab treatment in patients with metastatic urothelial carcinoma: A retrospective multicenter analysis. Oncology.

[12] Tamura D, Jinnouchi N, Abe M (2020). Prognostic outcomes and safety in patients treated with pembrolizumab for advanced urothelial carcinoma: experience in real-world clinical practice. Int. J. Clin. Oncol..

[13] Yamamoto Y, Yatsuda J, Shimokawa M (2021). Prognostic value of pre-treatment risk stratification and post-treatment neutrophil/lymphocyte ratio change for pembrolizumab in patients with advanced urothelial carcinoma. Int. J. Clin. Oncol..

[14] Azab BN, Bhatt VR, Vonfrolio S (2013). Value of the pretreatment albumin to globulin ratio in predicting long-term mortality in breast cancer patients. Am. J. Surg..

[15] Azab B, Kedia S, Shah N (2013). The value of the pretreatment albumin/globulin ratio in predicting the long-term survival in colorectal cancer. Int. J. Colorectal Dis..

[16] Liu J, Dai Y, Zhou F (2016). The prognostic role of preoperative serum albumin/globulin ratio in patients with bladder urothelial carcinoma undergoing radical cystectomy. Urol. Oncol..

[17] Liu Z, Huang H, Li S (2017). The prognostic value of preoperative serum albumin-globulin ratio for high-grade bladder urothelial carcinoma treated with radical cystectomy: A propensity score-matched analysis. J. Cancer Res. Ther..

[18] Niwa N, Matsumoto K, Ide H (2018). Prognostic value of pretreatment albumin-to-globulin ratio in patients with non-muscle-invasive bladder cancer. Clin. Genitourin Cancer.

[19] Zhang B, Yu W, Zhou LQ (2015). Prognostic significance of preoperative albumin-globulin ratio in patients with upper tract urothelial carcinoma. PLoS ONE.

[20] Xu H, Tan P, Ai J (2018). Prognostic impact of preoperative albumin-globulin ratio on oncologic outcomes in upper tract urothelial carcinoma treated with radical nephroureterectomy. Clin. Genitourin Cancer.

[21] Otsuka M, Kamasako T, Uemura T (2018). Prognostic role of the preoperative serum albumin: Globulin ratio after radical nephroureterectomy for upper tract urothelial carcinoma. Int. J. Urol..

[22] Fukushima H, Kobayashi M, Kawano K (2018). Prognostic value of albumin/globulin ratio in patients with upper tract urothelial carcinoma patients treated with radical nephroureterectomy. Anticancer Res..

[23] Omura S, Taguchi S, Miyagawa S (2020). Prognostic significance of the albumin-to-globulin ratio for upper tract urothelial carcinoma. BMC Urol..

[24] Pradere B, D'Andrea D, Schuettfort VM, UTUC collaboration (2020). Pre-therapy serum albumin-to-globulin ratio in patients treated with neoadjuvant chemotherapy and radical nephroureterectomy for upper tract urothelial carcinoma. World J. Urol..

[25] McShane LM, Altman DG, Sauerbrei W, Statistics Subcommittee of the NCI-EORTC Working Group on Cancer Diagnostics (2005). Reporting recommendations for tumor marker prognostic studies (REMARK). J. Natl. Cancer Inst..

[26] Matsuo S, Imai E, Horio M, Collaborators developing the Japanese equation for estimated GFR (2009). Revised equations for estimated GFR from serum creatinine in Japan. Am. J. Kidney Dis..

[27] Eisenhauer EA, Therasse P, Bogaerts J (2009). New response evaluation criteria in solid tumours: revised RECIST guideline (version 1.1). Eur. J. Cancer.

[28] Harrell FE, Lee KL, Mark DB (1996). Multivariable prognostic models: issues in developing models, evaluating assumptions and adequacy, and measuring and reducing errors. Stat. Med..

[29] He J, Pan H, Liang W (2017). Prognostic effect of albumin-to-globulin ratio in patients with solid tumors: A systematic review and meta-analysis. J. Cancer.

[30] Templeton AJ, McNamara MG, Šeruga B (2014). Prognostic role of neutrophil-to-lymphocyte ratio in solid tumors: a systematic review and meta-analysis. J. Natl. Cancer Inst..

