# Supplementary material for: Prognostic significance of the albumin-to-globulin ratio for advanced urothelial carcinoma treated with pembrolizumab: a multicenter retrospective study
Source: Sci Rep. 2021 Aug 2;11:15623. doi: 10.1038/s41598-021-95061-z (PMC8329063; doi:10.1038/s41598-021-95061-z)
Supplement: Supplementary file 1 — Supplementary Figure S1. [file 41598_2021_95061_MOESM1_ESM.pdf]

Supplementary Figure S1. ROC curve analyses of (A) AGR for PFS, (B) NLR for PFS, (C) AGR for CSS, (D) NLR for CSS, (E) AGR for OS, (F) NLR for OS. The optimal cutoff value of AGR or NLR was estimated through maximization of the Youden's index [Sensitivity − (1 − Specificity)] for each endpoint.

(A) ROC curve analysis of the AGR for PFS (no. of event: 111)

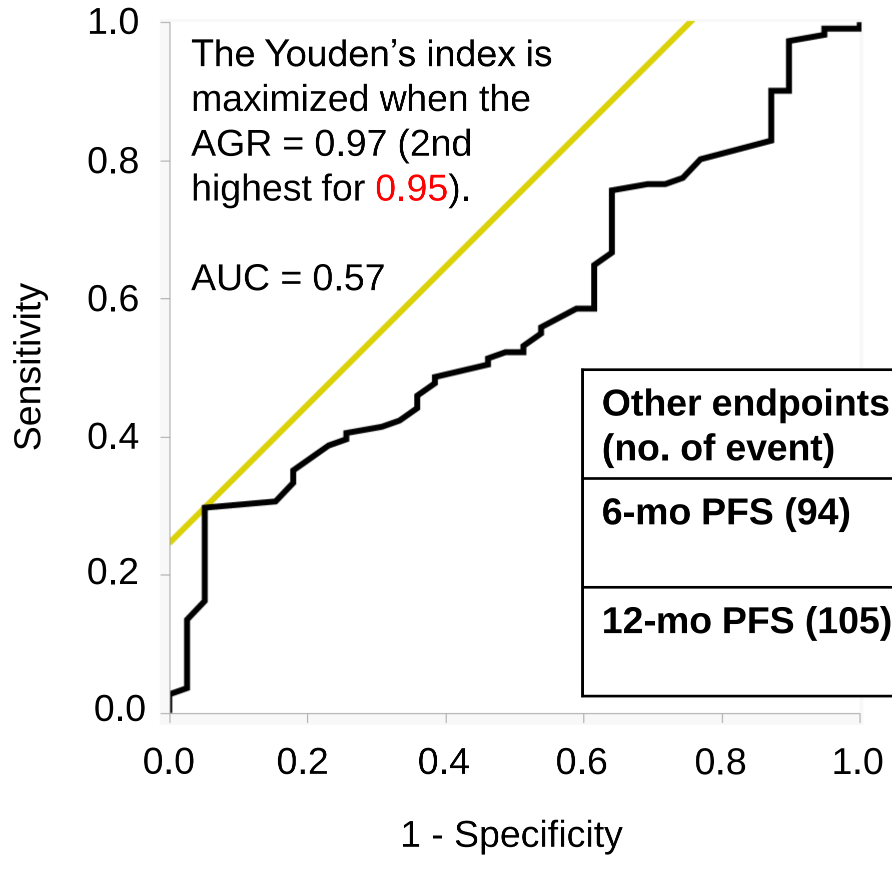

Other endpoints (no. of event)	AGR for the highest Youden's index
**6-mo PFS (94)**	0.97* (AUC = 0.56) *3rd highest for 0.95
**12-mo PFS (105)**	0.97* (AUC = 0.57) *2nd highest for 0.95

(B) ROC curve analysis of the NLR for PFS (no. of event: 111)

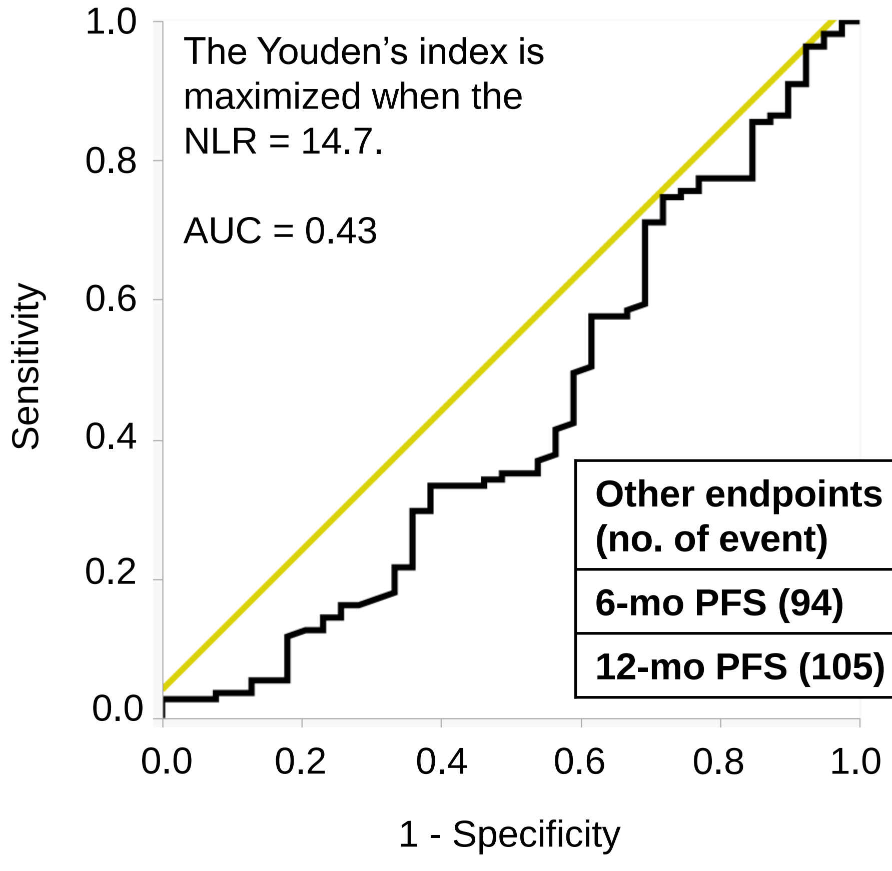

Other endpoints (no. of event)	NLR for the highest Youden's index
**6-mo PFS (94)**	3.02 (AUC = 0.55)
**12-mo PFS (105)**	3.02 (AUC = 0.46)

(C) ROC curve analysis of the AGR for CSS (no. of event: 75)

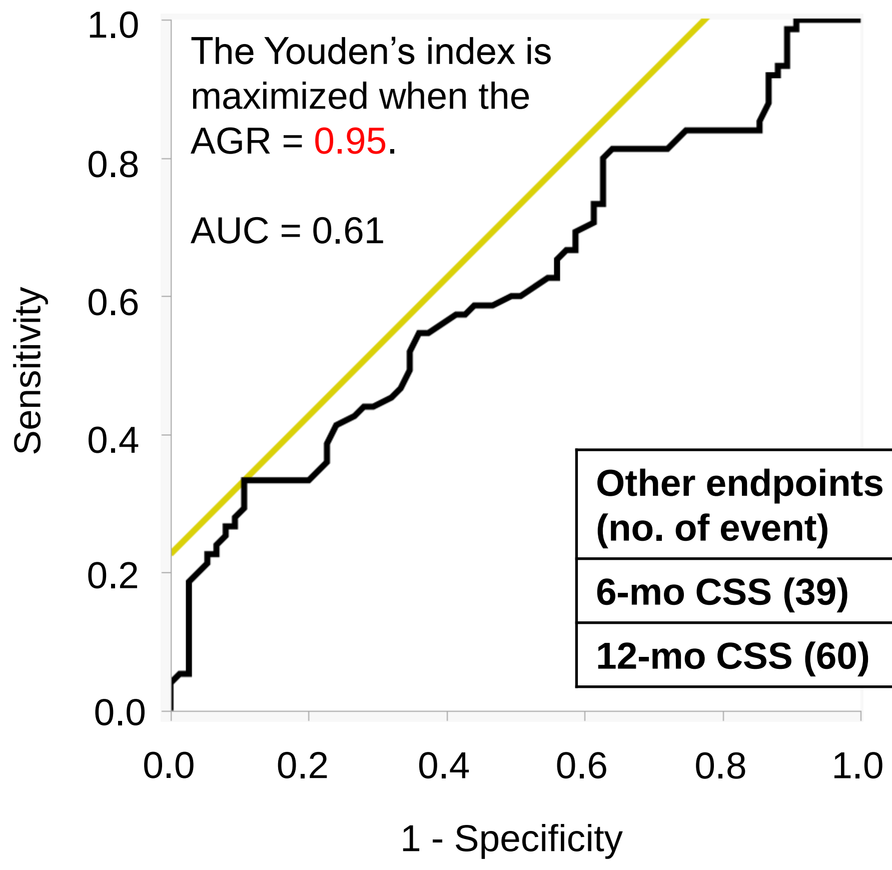

Other endpoints (no. of event)	AGR for the highest Youden's index
**6-mo CSS (39)**	0.95 (AUC = 0.72)
**12-mo CSS (60)**	0.95 (AUC = 0.61)

(D) ROC curve analysis of the NLR for CSS (no. of event: 75)

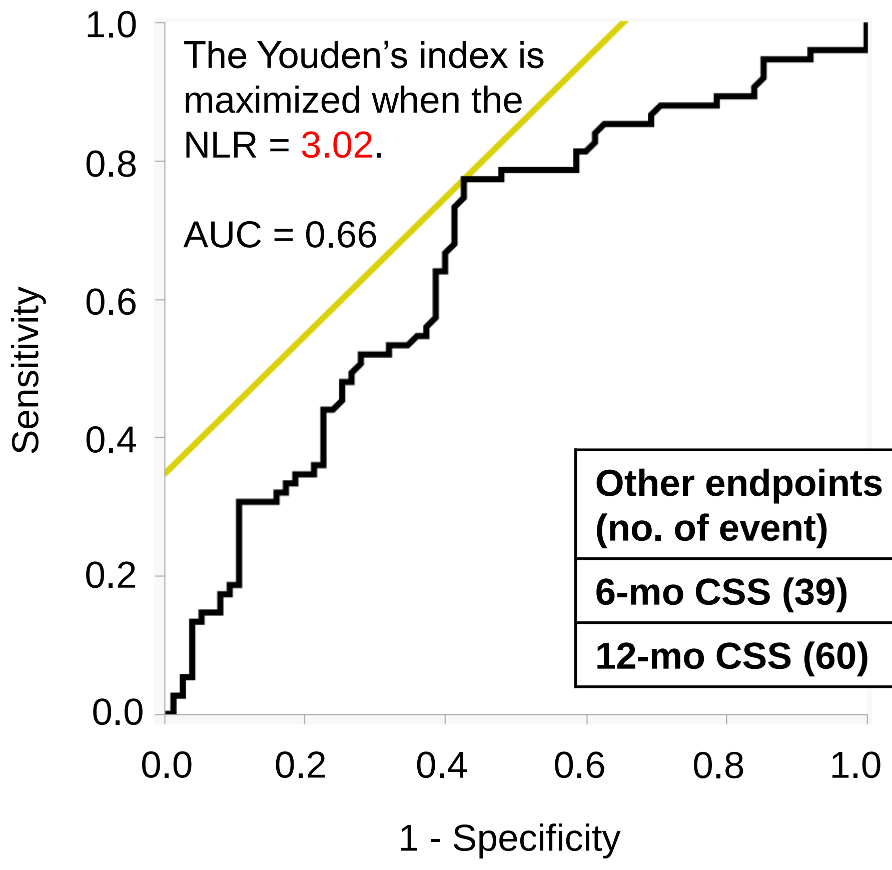

Other endpoints (no. of event)	NLR for the highest Youden's index
**6-mo CSS (39)**	4.29 (AUC = 0.77)
**12-mo CSS (60)**	3.02 (AUC = 0.66)

(E) ROC curve analysis of the AGR for OS (no. of event: 81)

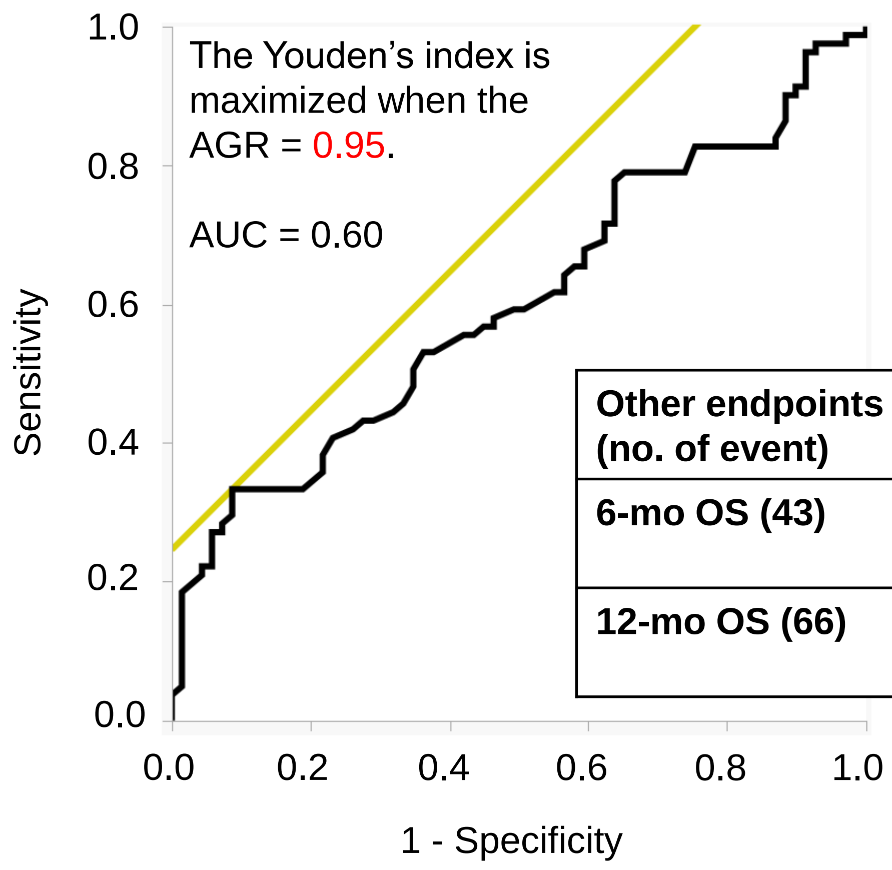

Other endpoints (no. of event)	AGR for the highest Youden's index
**6-mo OS (43)**	0.85* (AUC = 0.69) *2nd highest for 0.95
**12-mo OS (66)**	0.85* (AUC = 0.60) *2nd highest for 0.95

(F) ROC curve analysis of the NLR for OS (no. of event: 81)

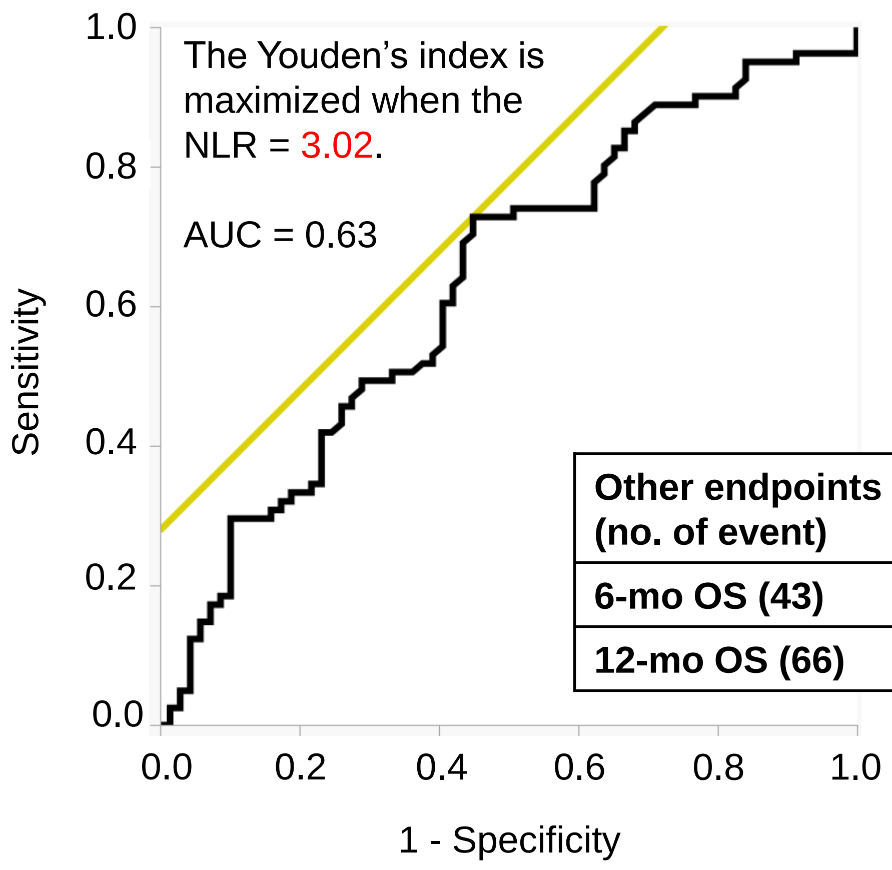

Other endpoints (no. of event)	NLR for the highest Youden's index
**6-mo OS (43)**	4.29 (AUC = 0.73)
**12-mo OS (66)**	5.79 (AUC = 0.63)
